# Engineering CRISPR/*Lb*Cas12a for highly efficient, temperature‐tolerant plant gene editing

**DOI:** 10.1111/pbi.13275

**Published:** 2019-10-31

**Authors:** Patrick Schindele, Holger Puchta

**Affiliations:** ^1^ Botanical Institute Karlsruhe Institute of Technology Karlsruhe Germany

**Keywords:** CRISPR, Cas12a, CRISPR, Cpf1, *Lb*Cas12a, temperature‐tolerant, gene editing

The discovery of the CRISPR/Cas9 system was a milestone for plant biotechnology enabling targeted mutagenesis with an unprecedented simplicity and accuracy. An abundant G‐rich protospacer‐adjacent motif (PAM) constitutes the sole requirement for Cas9 cleavage. Nevertheless, targeting of T‐rich sites in the genome such as non‐coding sequences remains challenging. The characterization of the CRISPR/Cas12a system provided a novel genome editing tool (Zetsche *et al.*, 2015). In contrast to Cas9, Cas12a utilizes a T‐rich PAM, expanding the scope of potential target sites. Moreover, DNA cleavage occurs distal to the PAM and produces staggered ends with 4–5 nt overhangs (Zetsche *et al.*, 2015). A variety of Cas12a orthologues have been identified which show solid genome editing activity in mammalian systems. For plants, Cas12a from *Lachnospiraceae bacterium ND2006* (*Lb*Cas12a) is the most widely used orthologue for targeted mutagenesis. However, a certain divergence in editing efficiency was observed between plant species and targets, mainly due to the reduced activity of the enzyme at lower temperatures, which are mandatory for plant cultivation (Bernabé‐Orts *et al.*, 2019; Lee *et al.*, 2019; Malzahn *et al.*, 2019). The Cas12a from *Acidaminococcus spec. BV3L6* (*As*Cas12a) shows an even higher temperature sensitivity. Only recently was a temperature‐insensitive variant of *As*Cas12a established, enhanced *As*Cas12a (en*As*Cas12a), showing on average a twofold increase in activity at lower temperatures compared with wild‐type *As*Cas12a in human cells (Kleinstiver *et al.*, 2019).

We were interested in obtaining temperature‐tolerant *Lb*Cas12a variants for application in plants. Since en*As*Cas12a was still outperformed by wild‐type *Lb*Cas12a in *in vitro* cleavage reactions at 32 and 25 °C (Kleinstiver *et al.*, 2019), application of en*As*Cas12a itself in plants did not seem promising. Therefore, we set out to use the knowledge obtained with *As*Cas12a to construct novel *Lb*Cas12a variants. Two variants were of special interest to us: The en*As*Cas12a variant performed best and harbours in total three amino acid substitutions E174R/S542R/K548R and the variant comprising the single substitution E174R that was identified as the main requirement for the enhanced efficiency at lower temperature (Kleinstiver *et al.*, 2019). We performed a protein sequence alignment with different Cas12a orthologues showing an especially high conservation of E174 and K548. We identified the respective amino acids in *Lb*Cas12a: E174, S542 and K548 in *As*Cas12a correspond to D156, G532 and K538 in *Lb*Cas12a, respectively. Two *Lb*Cas12a variants were engineered, an en*As*Cas12a‐analogue *Lb*Cas12a (en*Lb*Cas12a) harbouring three mutations, D156R/G532R/K538R and a temperature‐tolerant *Lb*Cas12a (tt*Lb*Cas12a) that harbours the single mutation D156R. Using a codon‐optimized Cas12a ORF (Wolter and Puchta, 2019), the variants were cloned under control of the constitutive Ubiquitin4‐2 promoter from parsley (Fauser *et al.*, 2014) along with ribozyme‐flanked crRNAs expressed by the Arabidopsis U6‐26 promoter. We selected five genomic targets and transformed the respective crRNAs with all three Cas12a variants as T‐DNAs into *Arabidopsis thaliana* using the floral dip method (Figure 1a). To exclude expression variations due to position effects, 10 different transgenic T1 plants were grown for two weeks at 22 °C and 28 °C, respectively, for each construct. After gDNA extraction, CAPS analysis was performed to evaluate the cleavage efficiencies. Gene editing was detected at all five targets for each of the variants. As an example, analysis of target two is shown in Figure 1b. The analysis revealed comparable low to medium activity levels for *Lb*Cas12a and en*Lb*Cas12a at 22 °C. By contrast, tt*Lb*Cas12a showed a tremendous increase in activity with most plants tested approaching complete gene editing. When the plants were grown at 28 °C, an increase in activity could be observed for *Lb*Cas12a, which is consistent with previous data (Malzahn *et al.*, 2019), and also en*Lb*Cas12a. However, in most cases the cleavage efficiencies at 28 °C were still far lower than that of tt*Lb*Cas12a at 22 °C, demonstrating the superiority of the temperature‐tolerance of this variant. Interestingly, incubation at 28 °C further enhanced tt*Lb*Cas12a activity. To validate the results obtained by CAPS analysis and enable quantitative comparison, TIDE analysis was conducted. The respective target sites were amplified for each individual transgenic T1 plant, subjected to Sanger sequencing, and the obtained data were subsequently processed for sequence decomposition. The calculated editing efficiencies are presented as the average values of the 10 plants in the table of Figure 1c, and the variation of the individual plants is documented as box plots in Figure 1d. tt*Lb*Cas12a showed the highest mean efficiencies at every single target (Figure 1c). On average, tt*Lb*Cas12a was between twofold to sevenfold more efficient at 22 °C compared to *Lb*Cas12a. The editing efficiencies of en*Lb*Cas12a were higher than that of the wild‐type enzyme, but only for some sites and still below the level of tt*Lb*Cas12a. These results differ from the data obtained for the engineered *As*Cas12a variants in human cells, where the analogue en*As*Cas12a showed higher efficiencies, indicating that the additional mutations interfere with the activity of *Lb*Cas12a. Incubation at 28 °C resulted in increased efficiencies for each variant at most of the target sites. However, *Lb*Cas12a and en*Lb*Cas12a were unable to reach levels of tt*Lb*Cas12a incubated at 22 °C for any of the edited sites (Figure 1c). If the variability of the different plants is taken into account (Figure 1d), a few more facts became apparent. There are sites such as target one that are almost completely resistant to editing by *Lb*Cas12a and en*Lb*Cas12a. Here, also increasing sample size does not raise the editing efficiency, and even tt*Lb*Cas12a shows low editing efficiencies across all samples. However, incubation at 28 °C helps to further increase editing efficiency with the temperature‐tolerant enzyme. For all the other targets even at 22 °C, tt*Lb*Cas12a achieves almost complete editing for single plants. tt*Lb*Cas12a dramatically outperforms *Lb*Cas12a especially at 22 °C, where almost all individual plants show much higher efficiencies for every single target. For some targets, en*Lb*Cas12a outperforms *Lb*Cas12a on the population level but not for others. At 28 °C, tt*Lb*Cas12a has uniform high efficiency across all plants for all targets tested. This is also demonstrated by the fact that a more detailed analysis of the sequencing chromatograms unveiled that for two plants edited by tt*Lb*Cas12a at 28 °C clonal biallelic mutations were already present at the T1 stage. Figure 1e shows a sequencing chromatogram from a biallelic mutant with a 13‐bp and a 14‐bp deletion, respectively. Analysis of the mutation profiles of plants edited by en*Lb*Cas12a or tt*Lb*Cas12a at 22 °C and 28 °C otherwise revealed the presence of chimeric mutations with patterns similar to that of the wild‐type enzyme.

**Figure 1:**
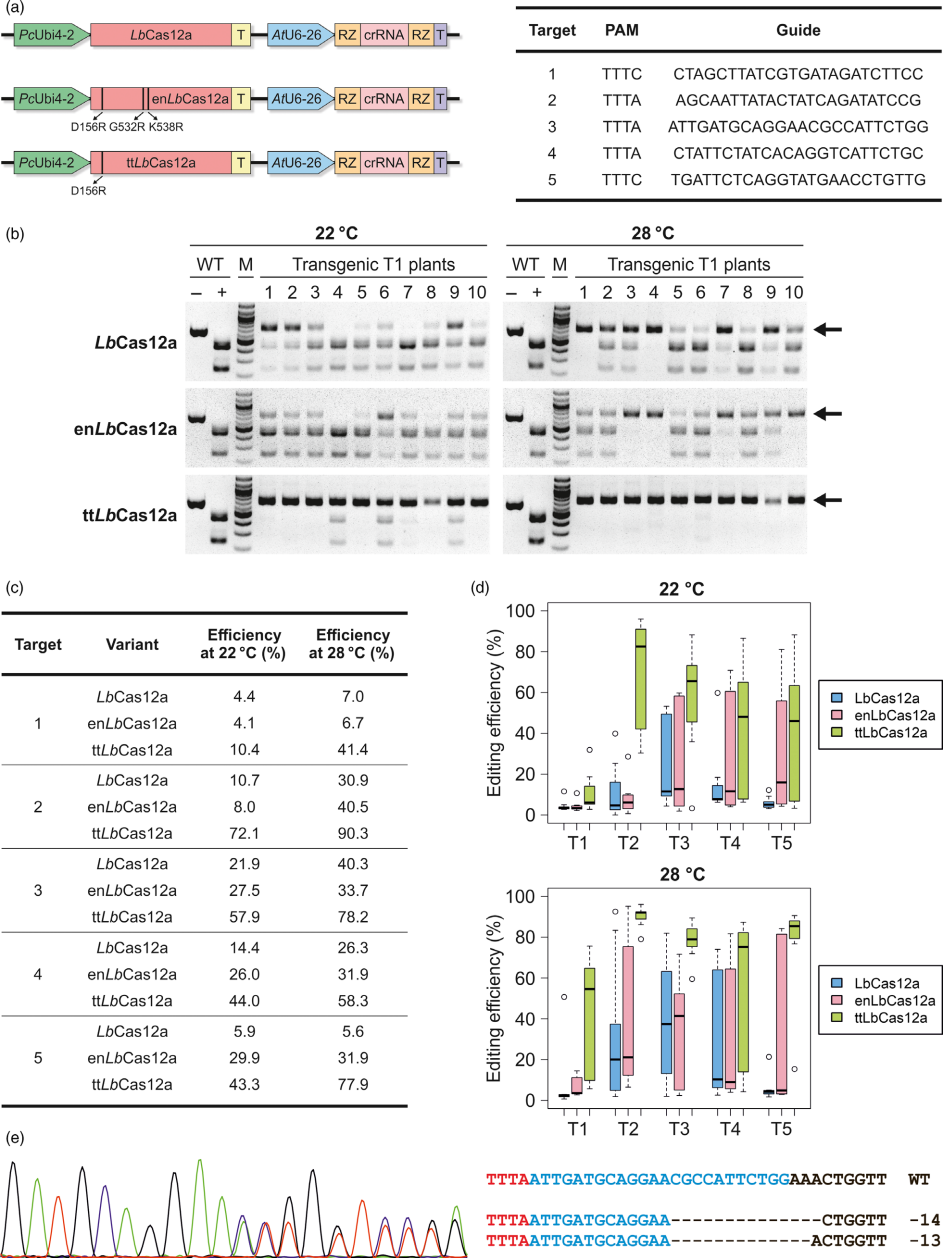
Engineered LbCas12a for temperature‐tolerant gene editing. (a) LbCas12a expression cassettes. The respective mutations are highlighted, and the target sites of the ECA3 locus are given. (b) CAPS analysis of 10 individual transgenic Arabidopsis T1 plants with a crRNA for target two comparing LbCas12a, enLbCas12a and ttLbCas12a. Genomic DNA of two‐week‐old wild‐type (WT) or transgenic plants (nos. 1‐10) grown at 22 °C or 28 °C, respectively, was isolated, the target site amplified by PCR and subjected to restriction digestion. M, marker; ‐, undigested PCR product; +, digested PCR product; Arrows indicate edited DNA. (c) Table showing the average editing efficiencies of 10 individual T1 plants transformed with the LbCas12a variants at the examined targets. Amplified targets (see (b)) were subjected to TIDE analysis. Efficiencies of the variants were determined for each of the tested targets at 22 °C and 28 °C, for each of the 10 individual transgenic plants. (d) Distribution of editing efficiencies of LbCas12a variants in individual plants. Illustrated are the editing efficiencies of each of the 10 transgenic plants at the tested targets (T1‐T5) for each of the variants at 22 °C and 28 °C. Data are presented as box plots, each box represents the 25th and 75th percentile, and the median is indicated by a black line. (e) Left: Sanger chromatogram of target three of a biallelic clonal T1 mutant plant transformed with ttLbCas12a and grown at 28 °C in comparison with wild‐type (WT); Right: Sequences of the mutated alleles are shown. The PAM is labelled in red, the guide labelled in blue. Deletions are indicated by hyphens.

Taken together, our results demonstrate that the two newly designed variants en*Lb*Cas12a and tt*Lb*Cas12a provide moderate and strong temperature‐tolerance, making especially the latter one a perfect tool for genome editing in plants that are cultivated at lower temperatures.

So far, several attempts have been conducted to establish a robust and highly efficient CRISPR/Cas12a system for plants. These include the incorporation of self‐cleaving ribozymes to mature crRNAs, the engineering of variants with altered PAM sites and the expression of Cas12a and crRNA in a single transcriptional unit (Tang *et al.*, 2017; Xu *et al.*, 2019; Zhong *et al.*, 2018). Depending on the target, it might be worth combining these approaches with tt*Lb*Cas12a to achieve even further improvements in DSB induction. While the repair of these DSBs by non‐homologous end joining leads to knockout edits, the novel Cas12a variants might also be helpful for achieving higher rates of gene targeting by homologous recombination (Wolter and Puchta, 2019). The novel Cas12a enzyme might also be applied as a DNA‐binding protein to improve Cas12a‐based applications in plants as has been observed for en*As*Cas12a in transcriptional regulation or base editing (Kleinstiver *et al.*, 2019).

## Author contributions

H.P. conceived the research. P.S. designed and executed the experiments. Both authors wrote the article.

## Conflict of Interest

The authors declare no conflict of interest.
